# The effect of *N*-arachidonoylethanolamide administration on energy and fat metabolism of early lactating dairy cows

**DOI:** 10.1038/s41598-023-41938-0

**Published:** 2023-09-06

**Authors:** Jessica Schwerdtfeger, Helga Sauerwein, Elke Albrecht, Gemma Mazzuoli-Weber, Dirk von Soosten, Sven Dänicke, Björn Kuhla

**Affiliations:** 1 Research Institute for Farm Animal Biology (FBN), Wilhelm-Stahl-Allee 2, 18196 Dummerstorf, Germany; 2 Institute of Physiology, Biochemistry and Animal Hygiene, Bonn University, Katzenburgweg 7-9, 53115 Bonn, Germany; 3 Institute for Physiology and Cell Biology, University of Veterinary Medicine Hannover, Bischofsholer Damm 15, 30173 Hannover, Germany; 4 Institute of Animal Nutrition, Friedrich-Loeffler-Institut (FLI), Federal Research Institute for Animal Health, Bundesallee 37, 38116 Brunswick, Germany

**Keywords:** Biochemistry, Physiology

## Abstract

The aim of the study was to investigate the effect of *N*-arachidonoylethanolamide (AEA), an endocannabinoid with orexigenic characteristics, on plasma endocannabinoid concentrations, feed intake, energy balance, lipomobilisation, and hepatic lipid metabolism of early-lactating dairy cows. The experiment involved 10 pairs of Holstein half-sibling cows (end of 2nd–3rd pregnancy). Half-sibs of each pair were randomly assigned to either AEA (n = 10) or control (CON) group (n = 10). From day 1 to 30 postpartum, the AEA group received 5 intraperitoneal injections per week of 3 µg/kg body weight AEA and the CON group 0.9% NaCl. In week 1–3 postpartum, AEA administration had no effect on dry matter intake, body weight, or lipomobilisation, but increased plasma triglyceride concentration on d 21 p.p. and mRNA abundances of genes related to hepatic triglyceride synthesis. In week 4 postpartum, the AEA group showed reduced feed intake and whole-body carbohydrate oxidation, but increased whole-body fat oxidation and hepatic lipid accumulation, likely as a result of a counter-regulatory leptin increase. In conclusion, the present study shows a tissue-specific AEA insensitivity and may point to a leptin-controlled regulation of the ECS in early-lactation.

## Introduction

The endocannabinoid system (ECS) is an important modulator of energy and fat metabolism in mammals. The endocannabinoids (EC) *N*-arachidonoylethanolamide (AEA), 2-arachidonoylglycerol (2-AG), docosahexaenoyl ethanolamide (DHEA), and eicosapentaenoyl ethanolamide (EPEA) bind to G-protein coupled receptors, like the cannabinoid receptor type 1 (CNR1)^1–3^ and the cannabinoid receptor type 2 (CNR2)^1,3,4^. In addition, AEA and 2-AG bind to the novel G-protein-coupled receptor 55 (GPR55)^5,6^. The structurally similar endocannabinoid-like compounds linoleoylethanolamide (LEA), palmitoylethanolamide (PEA), and oleylethanolamide (OEA) do not bind to CNR1 and CNR2, but share the same synthesis and degradation enzymes, specifically *N*-acyl-phosphatidylethanolamines hydrolysing phospholipase D (NAPELD) and fatty acid amide hydrolase (FAAH)^7,8^. Endocannabinoid-like compounds affect the EC tone by inhibiting hydrolysis or modulating the receptor binding^9^. Furthermore, the EC tone is also influenced by the anorexigenic hormone leptin^10^, which in turn reduces feed intake and regulates energy metabolism^11–13^.

The best-characterized endocannabinoid AEA is known to exert orexigenic effects. Peripheral and central administration of AEA increases short-term feed intake of mid- and late-lactating cows^14–16^. Furthermore, AEA is involved in the regulation of fat metabolism. Specifically, AEA administration or inhibition of EC degrading enzymes inhibits lipolysis^17^, promotes lipogenesis^18^, and decreases energy expenditure^19^, at least in non-ruminant species. In addition, administration of CNR1 agonists stimulates fatty acid synthesis in the liver of mice^20^. In late-lactating dairy cows, AEA administration influences whole-body metabolism by increasing carbohydrate oxidation (COX) and heat production (HP), while reducing fat oxidation (FOX)^15^. However, it is not known if AEA, would affect feed intake and metabolism of early-lactating dairy cows.

Early-lactating dairy cows experience tremendous changes in nutrient and energy requirements due to the onset of lactation. During the last week of pregnancy and the early postpartum (p.p.) period, cows reveal insufficient feed intake and enter a negative energy balance, resulting in the mobilisation of fat depots^21^. The mobilisation of body fat increases the plasma concentration of long-chain fatty acids (NEFA). Mobilized fatty acids serve as precursors for milk fat synthesis by the mammary gland^22^. In the liver, NEFA are oxidized or, if the oxidative capacity is exceeded, converted to ketone bodies thereby increasing the risk of metabolic disorders such as ketosis^23^. Another portion of NEFA entering the liver is re-esterified yielding triacylglycerols (TG)^23^. Hepatic TG are excreted to the circulation via very low density lipoproteins (VLDL), however, cattle have a slow VLDL secretion rate^24^, so excessive lipolysis results in hepatic lipid accumulation. A high liver fat content may decrease its metabolic function, which is related to depression of feed intake, productivity and health^25^. A high lipolysis rate after calving is associated with a higher AEA level and a higher mRNA abundance of the endocannabinoid receptors *CNR1* and *CNR2* in adipose tissue of cows^26^, but the lipolytic response of adipose tissues to an AEA stimulus is reduced during early lactation^27^. Moreover, a direct relationship between rising plasma AEA levels and the increase in feed intake during the early-lactation period of dairy cows has been reported^28^. Khan et al. demonstrated that prepartal energy intake alters the hepatic mRNA expression of genes related to the ECS in the p.p. period. Specifically, feeding a ration with an energy content exceeding the energy requirement during the dry period increased the expression of monoglyceride lipase (MAGL) and decreased the expression of HRAS-like suppressor family, member 5 (HRASLS5), synthesizing fatty acid amides^29^. These previous studies suggest that AEA is involved in the regulation of metabolic adaptation to early lactation. However, a study testing the cause-effect relationships of AEA administration to early-lactating dairy cows has not been performed.

We hypothesize that activation of the ECS by AEA increases feed intake but decreases fat mobilisation. To pursue this hypothesis, we aimed to elucidate the effect of intraperitoneal (i.p). AEA administration on dry matter intake, energy and fat metabolism of early-lactating dairy cows. By comparative analysis with data obtained from AEA-treated late-lactating cows, we additionally aimed at illuminating general principles and differences of AEA effects at different stages of lactation. Knowledge about the modulatory effect of AEA on feed intake, adipose tissue depots and liver fat in early-lactation could provide a better knowledge about metabolic adaptations p.p. and help to improve animal health.

## Results

### Body weight, feed intake, milk yield and energy balance

**Figure 1:**
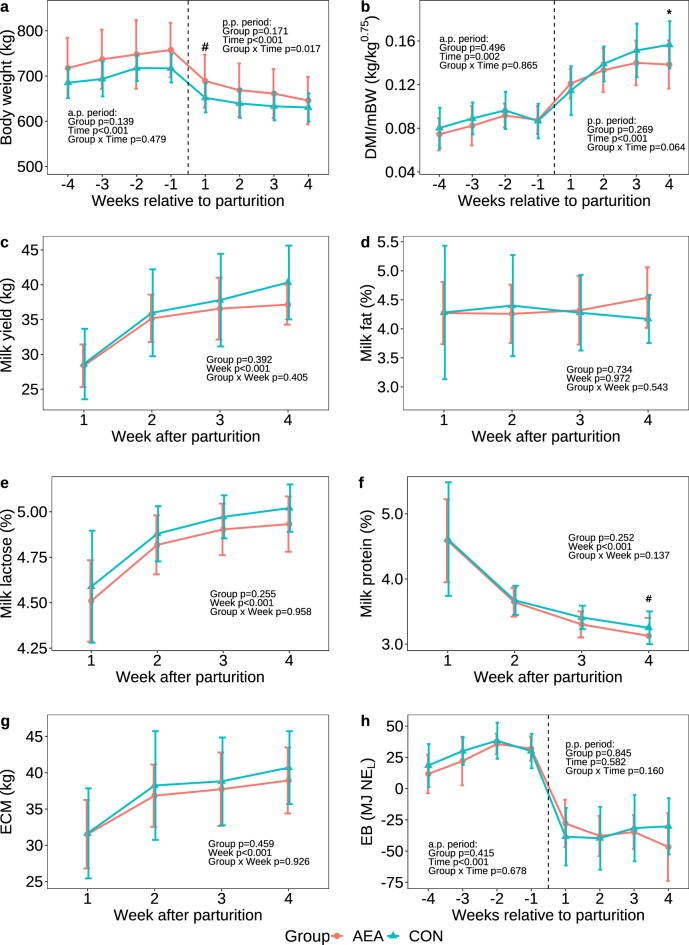
Body weight (**a**), dry mater intake normalized to metabolic body weight (DMI/mBW; **b)**, milk yield (**c**), milk fat concentration (**d**), milk lactose concentration (**e**), milk protein concentration (**f**), energy corrected milk yield (ECM; **g**), and energy balance (EB; **h**) of cows treated intraperitoneally with *N*-arachidonoylethanolamide (AEA, n = 10) or NaCl (CON, n = 10) postpartum. Data are presented as means ± SD; # *P* < 0.1, *P < 0.05.

The body weight (BW) was not different between AEA and CON cows in the antepartum (a.p.) and p.p. period, although it tended to be higher in AEA cows in week 1 p.p. (*P* = 0.080; Fig. 1a). The dry matter intake (DMI) normalized to metabolic body weight (mBW) did not differ between groups in the a.p. period and from week 1–3 p.p., but in week 4 p.p., the AEA group revealed 1.13-fold lower intake (*P* = 0.013) as compared to the CON group (Fig. 1b). During lactation, milk yield, milk fat, milk lactose, and milk protein concentrations (Fig. 1c–f) did also not differ between groups (*P* > 0.1). However, in week 4 p.p. milk protein concentration tended to be lower in AEA than CON cows (*P* = 0.056, Fig. 1f). In addition, milk urea concentrations (Supplementary Fig. 1), energy corrected milk yield (ECM) (Fig. 1g) and energy balance were not different between groups (*P* > 0.1, Fig. 1h).

### Body anatomy and body condition

**Figure 2:**
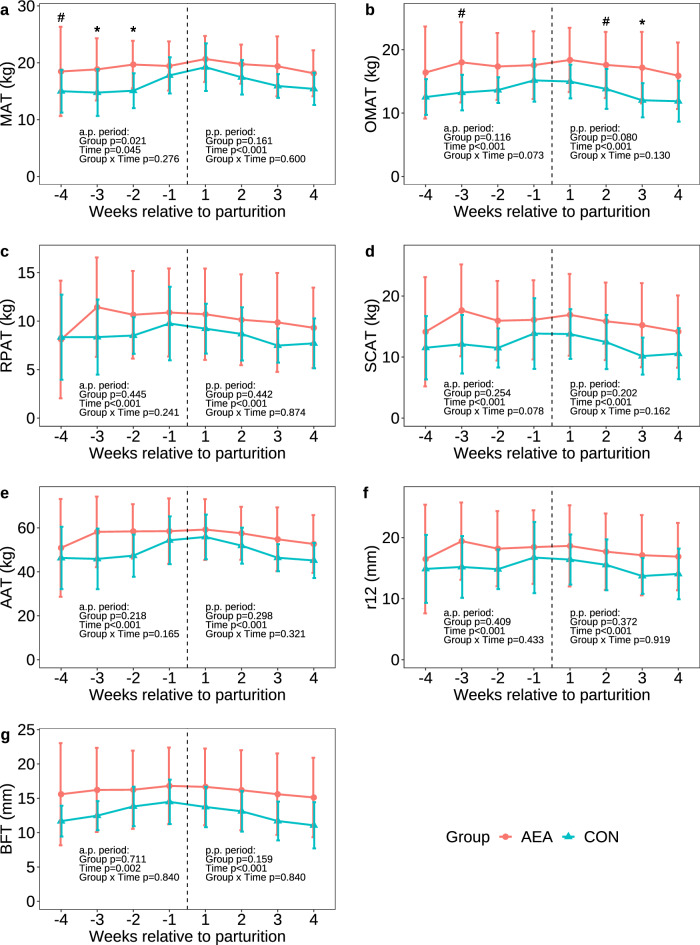
Amount of estimated mesenteric adipose tissue (MAT; **a**), omental adipose tissue (OMAT; **b**), retroperitoneal adipose tissue (RPAT; **c**), subcutaneous adipose tissue (SCAT; **d**), total abdominal adipose tissue (AAT; **e**), thickness of the fat layer over the 12th rib (r12; **f**), and back fat thickness (BFT; **g**) of cows treated intraperitoneally with *N*-arachidonoylethanolamide (AEA, n = 10) or NaCl (CON, n = 9) postpartum. Data are presented as means ± SD; # *P* < 0.1, *P < 0.05, **P < 0.01.

The heart girth (Supplementary Fig. 2a) and body condition score (BCS) (Supplementary Fig. 2b) did not differ between groups, in neither the a.p. nor p.p. period, except for the heart girth in week 4 p.p., which tended to be higher in AEA cows. However, AEA cows tended to have higher amounts of estimated mesenteric adipose tissue in week 4 a.p., (*P* = 0.083; Fig. 2a) and more mesenteric adipose tissue in week 3 and 2 a.p. (*P* < 0.050; Fig. 2a). In addition, AEA cows tended to have more omental adipose tissue in week 3 a.p. and week 2 p.p. (*P* < 0.100) and had more omental adipose tissue in week 3 p.p. (*P* = 0.040; Fig. 2b). By contrast, the amounts of retroperitoneal adipose tissue, subcutaneous adipose tissue, and total abdominal adipose tissue (Fig. 2c–e) were not different between groups, but subcutaneous adipose tissue tended to be affected by a group by time interaction before parturition (*P* = 0.078). The thickness of the fat layer over the 12th rib and the back fat thickness were not affected by group before and after parturition (*P* > 0.1; Fig. 2f,g). However, mBW declined during the first 4 weeks of the p.p. period more in AEA than CON cows (group x time interaction: *P* = 0.021; Supplementary Fig. 2c).

### Plasma EC and NAE concentrations

**Figure 3:**
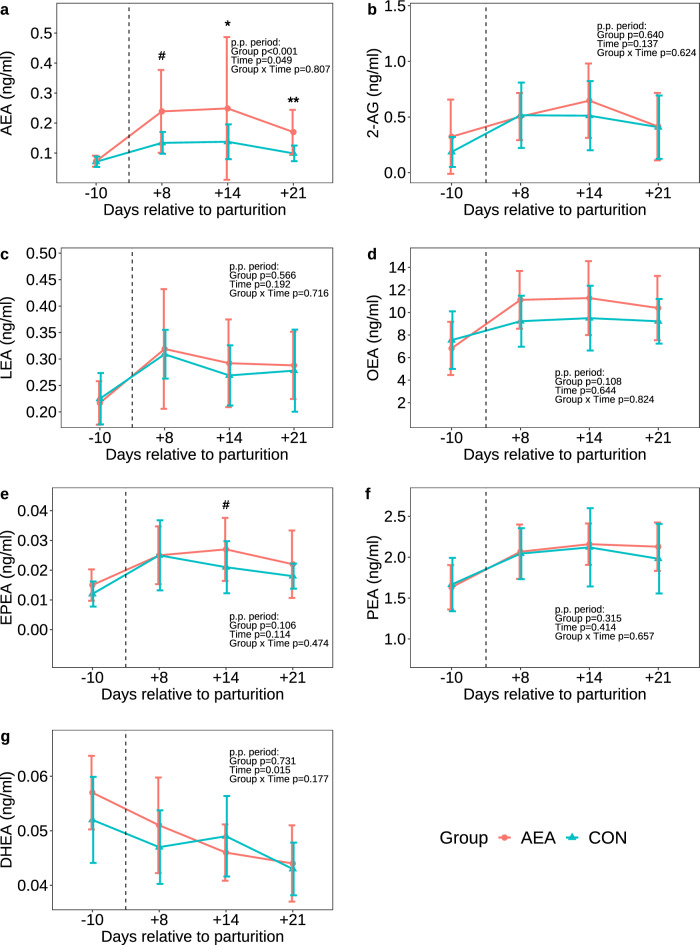
Plasma concentrations of *N*-arachidonoylethanolamine (AEA; **a**), 2-arachidonoylglycerol (2-AG; **b**), *N*-linoleoylethanolamide (LEA; **c**), oleoylethanolamide (OEA; **d**), eicosapentaenoyl ethanolamide (EPEA; **e**), *N*-palmitoylethanolamide (PEA; **f**), and docosahexaenoyl ethanolamide (DHEA; **g**) in cows treated intraperitoneally with *N*-arachidonoylethanolamide (AEA, n = 10) or NaCl (CON, n = 10) postpartum. Data are presented as means ± SD; # *P* < 0.1 *P < 0.05, **P < 0.01.

Next, we examined the effect of postparturient i.p. AEA administration on EC concentrations in the circulation. Before start of AEA administration, i.e. on day (d) 10 a.p., plasma AEA concentrations did not differ between groups (Fig. 3a). After start of the AEA injection series, plasma AEA concentrations were 1.72- to 1.81-fold higher in AEA than CON cows (*P* < 0.05). However, the plasma concentrations of 2-AG, LEA, OEA, PEA and DHEA were not affected by AEA treatment at any time p.p. (Fig. 3b–g), except for EPEA, which tended to be higher in the AEA group on d 14 p.p. (*P* = 0.094, Fig. 3e). In addition, plasma AEA, 2-AG, LEA, OEA, EPEA and PEA concentrations increased, while the DHEA concentration decreased in both groups from the a.p. to the p.p. period (Fig. 3g).

### Plasma metabolite concentrations

**Figure 4:**
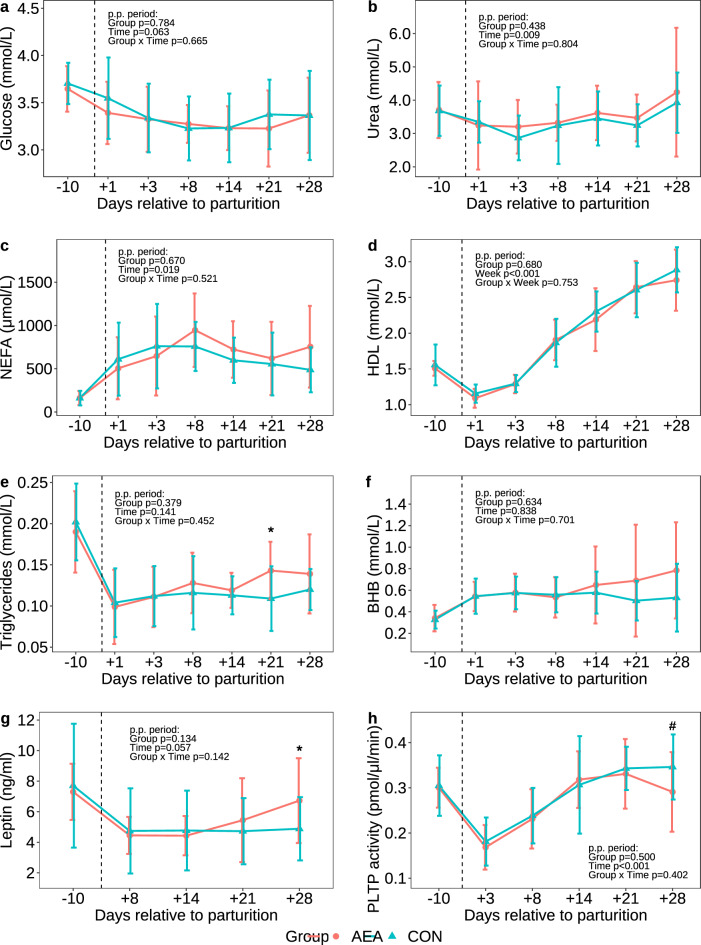
Plasma concentrations of glucose (**a**), urea (**b**), nonesterified fatty acids (NEFA; **c**), high-density lipoprotein cholesterol (HDL; **d**), triglycerides (**e**), β-hydroxybutyrate (BHB; **f**), leptin (**g**), and the activity of phospholipid transfer protein (PLTP; **h**) of cows treated intraperitoneally with *N*-arachidonoylethanolamide (AEA, n = 10) or NaCl (CON, n = 10) postpartum. Data are presented as means ± SD; # *P* < 0.1 *P < 0.05.

Plasma glucose and urea concentrations, NEFA, high-density lipoprotein cholesterol and TG were comparable between groups (*P* > 0.1; Fig. 4a–e), although AEA cows had higher TG concentrations on d 21 p.p. (*P* = 0.040, Fig. 4e). Similarly, plasma β-hydroxybutyrate and leptin concentrations, as well as the phospholipid transfer protein (PLTP) activity did not differ between groups (*P* > 0.1; Fig. 4f–h), except on d 28 after calving, on which AEA cows had a higher leptin concentration (*P* = 0.026, Fig. 4g) and tended to have a lower PLTP activity compared to CON cows (*P* = 0.087, Fig. 4h).

### Ex vivo adipose tissue lipolysis

We observed no group differences in the amount of glycerol released of from cultivated subcutaneous adipose tissue after stimulation with norepinephrine, neither a.p. nor p.p. (Supplementary Fig. 3).

### Hepatic ECS and fat metabolism

**Figure 5:**
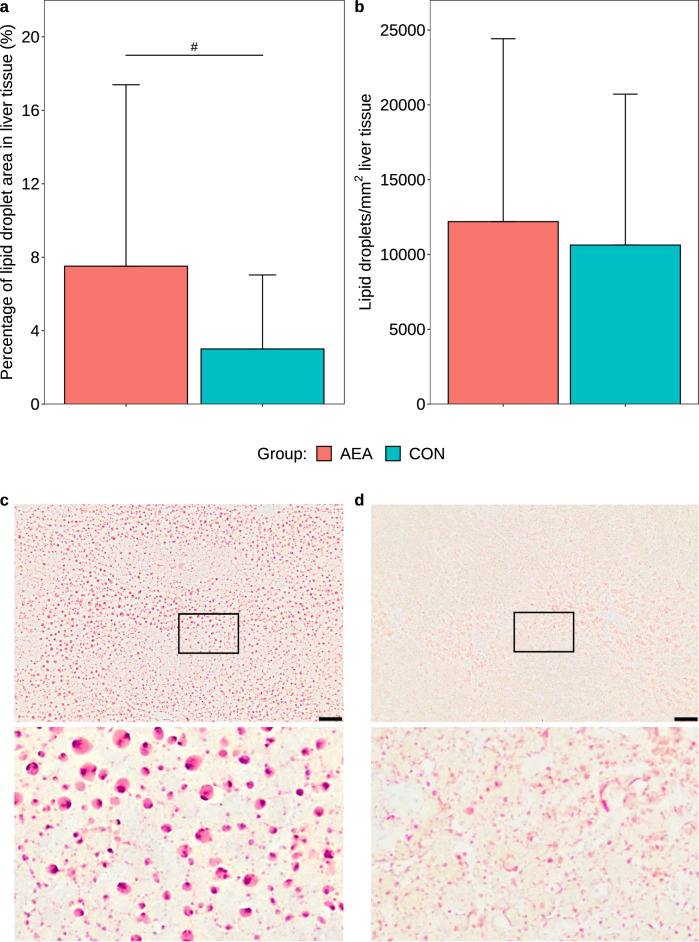
Percentage of the lipid droplet area of the total area in the liver tissue (**a**), and number of lipid droplets per mm^2^ in liver tissue (**b**) of cows treated intraperitoneally with *N*-arachidonoylethanolamide (AEA, n = 9) or NaCl (CON, n = 10) postpartum. Data are presented as means ± SD; # *P* < 0.1, * *P* < 0.05. Oil red O staining of liver sections from the AEA (**c**), and the CON group (**d**). The black framed areas from the top images are shown enlarged at the bottom. Scale bar represents 50 µm.

Hepatic fat deposition was quantified by the analysis of Oil Red O stained liver sections (Fig. 5c,d). The percentage of the lipid droplet area of the total area in the liver tended to be higher in the AEA compared to the CON group (*P* = 0.054, Fig. 5a), however, the number of lipid droplets per mm^2^ liver tissue on d 30 p.p. did not differ between the groups (*P* = 0.401, Fig. 5b).

In regard to hepatic fat metabolism, diacylglycerol O-acyltransferase 1 (*DGAT1)* and diacylglycerol O-acyltransferase 2 (*DGAT2)* expression were lower in AEA compared to CON cows (*P* < 0.05, Table 1), whereas the transcript levels of ATP binding cassette subfamily a member (*ABCA1),* apolipoprotein B *(APOB100),* microsomal triglyceride transfer protein (*MTTP),* sterol regulatory element binding transcription factor 1 (*SREBF1*) and peroxisome proliferator activated receptor alpha (*PPARA*) were comparable between the groups on day 16 p.p.

**Table 1:** Relative mRNA abundances in the liver of cows treated intraperitoneally with *N*-arachidonoylethanolamide (AEA, n = 9) or NaCl (CON, n = 10) postpartum (p.p.). Tissue biopsies were obtained on d 25 antepartum (a.p.), d + 16 p.p. and on d 30 p.p. after slaughter and are presented as means ± SD.

Gene	Sample time point	AEA	CON	*P* value
DGAT1	25 d a.p	1.18 ± 0.80	1.34 ± 1.19	0.286
	16 d p.p	0.94 ± 0.93	1.57 ± 0.97	0.046
	30 d p.p	1.37 ± 0.85	1.25 ± 0.99	0.778
DGAT2	25 d a.p	1.00 ± 0.53	1.38 ± 1.10	0.957
	16 d p.p	0.84 ± 0.56	1.46 ± 0.80	0.049
	30 d p.p	1.16 ± 0.59	1.12 ± 0.87	0.879
CNR1	25 d a.p	1.05 ± 0.63	1.42 ± 0.20	0.997
	16 d p.p	0.93 ± 0.81	1.51 ± 0.08	0.103
	30 d p.p	1.16 ± 0.68	1.43 ± 1.24	0.584
GPR55	25 d a.p	1.00 ± 0.73	1.45 ± 1.34	0.678
	16 d p.p	0.95 ± 0.74	1.52 ± 0.93	0.091
	30 d p.p	1.15 ± 0.58	1.20 ± 0.96	0.955
FAAH	25 d a.p	0.78 ± 0.20	0.73 ± 0.13	0.620
	16 d p.p	1.38 ± 0.44	1.27 ± 0.37	0.437
	30 d p.p	1.08 ± 0.15	0.94 ± 0.14	0.028
NAPEPLD	25 d a.p	0.82 ± 0.28	0.93 ± 0.35	0.516
	16 d p.p	1.11 ± 0.15	1.25 ± 0.22	0.100
	30 d p.p	0.99 ± 0.18	1.03 ± 0.16	0.578
ABCA1	25 d a.p	1.07 ± 0.30	1.03 ± 0.48	0.678
	16 d p.p	1.07 ± 0.31	1.07 ± 0.40	0.719
	30 d p.p	0.93 ± 0.25	1.15 ± 0.49	0.195
APOB100	25 d a.p	0.90 ± 0.19	0.96 ± 0.20	0.392
	16 d p.p	1.11 ± 0.21	1.06 ± 0.14	0.519
	30 d p.p	1.02 ± 0.14	1.01 ± 0.16	0.925
MTTP	25 d a.p	0.98 ± 0.21	0.93 ± 0.21	0.662
	16 d p.p	1.09 ± 0.17	1.05 ± 0.17	0.630
	30 d p.p	0.94 ± 0.10	1.06 ± 0.14	0.061
SREBF1	25 d a.p	0.99 ± 0.13	1.02 ± 0.28	0.815
	16 d p.p	1.06 ± 0.23	1.05 ± 0.29	0.985
	30 d p.p	1.05 ± 0.21	1.00 ± 0.27	0.760
PPARa2	25 d a.p	1.57 ± 0.84	1.64 ± 1.61	0.993
	16 d p.p	2.34 ± 1.32	1.63 ± 1.42	0.157
	30 d p.p	2.53 ± 1.34	1.87 ± 1.79	0.340

*DGAT1/2* diacylglycerol O-acyltransferase 1/2; *ABCA1* ATP binding cassette subfamily a 1; *APOB100* apolipoprotein B; *MTTP* microsomal triglyceride transfer protein; *SREBF1* sterol regulatory element binding transcription factor 1; *PPARA* peroxisome proliferator activated receptor alpha, *CNR1* cannabinoid receptor 1; *GPR55* G protein-coupled receptor 55; *FAAH* fatty acid amide hydrolase; *NAPEPLD N*-acyl phosphatidylethanolamine phospholipase D.

Before parturition, the hepatic mRNA expression of genes involved in the ECS (*CNR1, GPR55, FAAH, NAPEPLD*) were not different between groups (*P* > 0.1, Table 1). In addition, genes related to fat metabolism *(ABCA1, APOB100, MTTP, SREBF1, PPARA, DGAT1, DGAT2)* did also not differ between groups. On day 16 of the p.p. period, the *GPR55* expression level tended to be lower in the AEA compared to the CON group (*P* = 0.091), however, AEA administration had no effect on *CNR1*, *FAAH* and *NAPEPLD* mRNA expression levels (*P* > 0.1, Table 1).

The mRNA expression analyses in liver obtained after slaughter revealed a higher *FAAH* abundance in the AEA compared to the CON group (*P* = 0.028, Table 1). In addition, *MTTP* mRNA abundance tended to be lower in AEA cows (*P* = 0.061), whereas AEA administration did not affect the abundance of genes involved in the ECS.

### Mammary gland ECS and fat metabolism

The relative mRNA expression levels of genes related to ECS and fat metabolism were not different between the AEA and CON group in the mammary gland p.p., however, acetyl-CoA carboxylase alpha (*ACC1*) mRNA expression was lower in the AEA than the control group (*P* = 0.031, Table 2).

**Table 2:** Relative mRNA abundances of genes related to the endocannabinoid system and fat metabolism in the mammary gland of cows treated intraperitoneally with *N*-arachidonoylethanolamide (AEA, n = 9) or NaCl (CON, n = 10) on d 30 postpartum (p.p.). Tissue samples were obtained on d 30 p.p. and data are shown as means ± SD.

Gene	AEA	CON	*P* value
*CNR1*	1.03 ± 0.50	1.18 ± 0.34	0.465
*ACC1*	0.96 ± 0.16	1.09 ± 0.21	0.031
*DGAT1*	1.06 ± 0.60	1.23 ± 0.39	0.467
*DGAT2*	1.13 ± 0.62	1.16 ± 0.17	0.848
*FASN*	0.97 ± 0.25	1.09 ± 0.34	0.402
*SCD*	0.94 ± 0.21	1.14 ± 0.38	0.137
*SREBF1*	1.03 ± 0.35	1.00 ± 0.15	0.774

*ACC1* acetyl-CoA carboxylase alpha; *CNR1* cannabinoid receptor 1; *DGAT1/2* diacylglycerol O-acyltransferase 1/2; *FASN* fatty acid synthase; *SCD* stearoyl-CoA desaturase; *SREBF1* sterol regulatory element binding transcription factor 1.

### Short-term feed intake and energy metabolism in the fourth week postpartum

**Figure 6:**
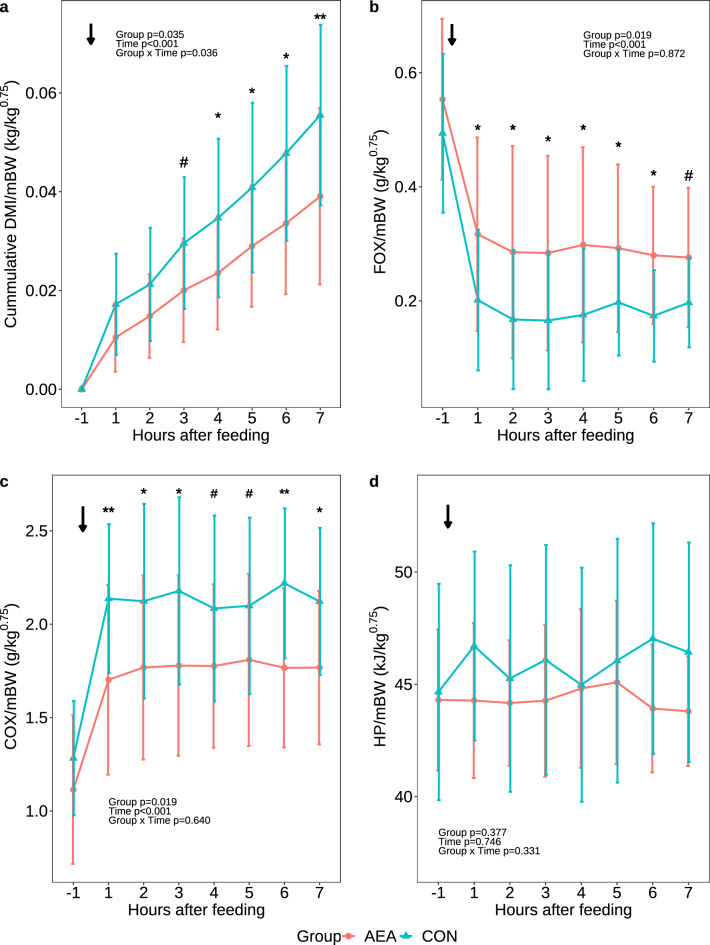
Cumulative DMI normalized to metabolic bodyweight (DMI/mBW; **a**), hourly fat oxidation (FOX) normalized to metabolic bodyweight (FOX/mBW; **b**), carbohydrate oxidation (COX) normalized to metabolic bodyweight (COX/mBW; **c**), and heat production (HP) normalized to metabolic bodyweight (HP/mBW; **d**) of cows treated intraperitoneally (i.p.) with *N*-arachidonoylethanolamide (AEA, n = 8) or NaCl (CON, n = 9) postpartum. The arrow indicates the time of i.p. injection (0700 h). Data are presented as means ± SD; # *P* < 0.1 *P < 0.05, **P < 0.01.

On d 27 p.p., cows were transferred to a respiration chamber to measure the short-term responses of feed intake and energy metabolism after AEA administration. Cumulative DMI normalized to mBW was not different in the first two h after feeding, but tended to be higher in the CON group 3 h after feeding (*P* = 0.076) and was higher than in the AEA group 4–7 h after feeding start (*P* < 0.05; Fig. 6a). Moreover, FOX normalized to mBW decreased more, and COX normalized to mBW increased more in CON cows to differ from AEA cows after feeding (*P* = 0.019, Fig. 6b,c). However, HP/mBW did not differ between groups at any time post feeding (*P* > 0.1, Fig. 6d).

## Discussion

In the present study, repeated administration of AEA was applied to increase plasma AEA concentrations in half-sib early-lactating cows. Activation of the ECS was expected to increase dry matter intake and decrease lipolysis, as already described in studies with rodents^17,30–34^.

### Effect of AEA administration on the plasma concentration of endocannabinoids and endocannabinoid-like compounds

In our study, we were able to successfully increase the plasma AEA concentration in early-lactation by repeated daily i.p. injections of 3 µg AEA/kg BW in the first four weeks of lactation. The treatment was specific to AEA, because it did not affect the plasma concentration of other endocannabinoids or endocannabinoid-like compounds, except for EPEA, which tended to be elevated on d 14 p.p. To date, no study has investigated, if AEA administration influences plasma EPEA concentration. In rodents, diet composition can affect EPEA levels in plasma and adipose tissue^35^, but all animals in our study were fed the same diet and consumed comparable amounts of feed. Thus, it remains elusive whether AEA administration or an unknown influencing factor caused this temporary increase in EPEA. The latter is a CNR1 and CNR2 agonist^3^ and possess anti-inflammatory properties^36^; however, further studies are needed to evaluate the role of EPEA on early-lactating cows.

### Effect of AEA on energy balance and hepatic gene expression in the first three weeks after parturition

Because we observed major differences in energy metabolism between weeks 1–3 p.p. and weeks 4–5 p.p., and that the transition period is defined 3 weeks before until 3 weeks after calving^37^, the discussion distinguishes between these 2 p.p. periods. Despite elevated plasma AEA concentrations, we did not observe an increase in feed intake during the first three weeks after calving in the AEA group. This result is in contrast to a study in rodents, which used a comparable low AEA dosage, but non-lactating animals^31^. Furthermore, AEA administration at similar dosage increased feed intake in mid- and late-lactation cows, although this effect was limited to 1 h after i.p. injection and to 10 h after intracerebroventricular injection^14,15,28^. The authors of these studies explained this short-term effect with the short half-life of AEA, because it is rapidly inactivated by re-uptake and degrading enzymes^38^. It is conceivable that the AEA treatment in the present study also caused a short-term increase in feed intake but this effect could not be detected on the daily basis. Furthermore, it has to be taken into account that the endocrine status differs substantially between early, late and non-lactating animals. Another important factor is that the sensitivity towards various hormones regulating feed intake, i.e., leptin, and perhaps also AEA, is diminished in early-lactation. Finally, we cannot exclude that AEA administrations on only 5 days a week affected the results.

Administration of AEA had also no effect on milk yield, ECM yield, and milk constituents, which together with the unaltered feed intake resulted in a comparable energy balance between the groups in the first 3 weeks p.p. This finding is underlined by comparable plasma NEFA and β-hydroxybutyrate concentrations between the groups. A previous study reported also no changes in plasma NEFA and β-hydroxybutyrate concentrations after i.p. AEA administration to late-lactating cows^15^. However, a reduction in milk yield after intracerebroventricular injection was observed in mid- and late-lactating cows^16^.

Previous studies in rodents, humans, and cows reported the involvement of the ECS in the regulation of lipolysis^17,27,39^. In early-lactating cows, fat mobilisation occurs due to the negative energy balance, resulting in a loss of adipose tissue mass and increase of plasma NEFA concentration. To our knowledge, the present study is the first to investigate the influence of AEA administration on fat depots in vivo. In our study, we did not observe a clear effect of AEA treatment on various fat depots and fat layer thicknesses, although the amount of omental adipose tissue tended to be higher in the AEA group in week 2 p.p. and was higher in week 3 p.p. One explanation could be that omental fat depots have a different sensitivity towards AEA than the other tissue types investigated, and this assumption is supported by the finding that various adipose tissue types reveal different responsiveness to lipolytic signals^40,41^. However, mesenteric fat depots were greater already in the a.p. period and could have affected cow’s metabolism p.p. A further limitation of the study is that we had no information about milk yield in the previous lactation which might have influenced body fat accretion a.p.

The plasma NEFA concentration is a marker for fat mobilisation^42^. The plasma NEFA concentration were not different between the first 3 weeks p.p. Moreover, we did not observe any effect on the norepinephrine-stimulated in vitro lipolysis in week 2 p.p. This finding is consistent with a previous study showing no changes in isoproterenol-induced lipolysis after CNR1 receptor agonist administration^27^. Overall, our and earlier results indicate that AEA has no or only a limited effect on lipomobilisation in early-lactation. This finding supports the hypothesis proposed by Myers et al.^27^, who suggested a resistance to ECS activation in adipose tissue of early-lactating cows^27^. The authors reported no effect of CNR1 receptor activation on the lipolysis rate in adipose tissue explants from cows obtained 1–3 weeks p.p.^27^.

Lipid mobilisation and the resulting increased influx of NEFA into the liver can exceed the hepatic capacity for oxidizing fatty acids, thus leading to lipid accumulation in liver of cows during early-lactation^43^. In rodents, the activation of the ECS increases fatty acid synthesis and lipid deposition in the liver^20^. In the present study, we showed that i.p. AEA administration decreased hepatic *GPR55* mRNA expression. Downregulation of G-protein coupled receptors is induced, among others, by prolonged exposure to an agonist^44,45^. Therefore, the diminished *GPR55* expression in the present study is likely due to chronic exposure of AEA, a *GPR55* agonist^46^. To date, the physiological role of *GPR55* in the liver of ruminants is unresolved. In humans and rodents, *GPR55* is involved in the regulation of hepatic lipid metabolism^47,48^ and insulin signalling^49^. Thus, the lower *GPR55* mRNA abundance suggests that administered AEA alters hepatic lipid metabolism through *GPR55* in cattle. Indeed, AEA treated cows showed reduced *DGAT1* and *DGAT2* mRNA abundance. Inhibition of *DGAT1* has been shown to result in less lipid droplet accumulation and TG concentration in primary calf hepatocytes after incubation with fatty acids. Thus, repeated AEA treatment may limit TG accumulation in liver and thus the progression of fatty liver in week 3 p.p.

In addition, we observed higher plasma TG concentration on d 21 p.p. in the AEA group. In mice, activation of the ECS resulted in higher plasma TG and cholesterol levels by impairing apolipoprotein E-mediated clearance^50^. In contrast to the finding in mice, we did not observe increased plasma cholesterol levels. In general, the increase in plasma TG can be induced either by increased synthesis of the liver or by decreased utilization in the mammary gland. Due to the comparable milk yield and milk fat content between the cow groups in week 3 p.p., a change in TG utilization for milk fat synthesis is rather unlikely. The synthesis and secretion of TG in cows occurs primarily in the liver through the re-esterification of NEFA and subsequent TG export as VLDL. The increase in plasma TG concentrations in week 3 p.p. might be due to an increased VLDL synthesis and secretion. Yet, we observed no difference in the mRNA expression of *APOB100* and *MTTP*, both genes involved in VLDL assembly^51^, but we cannot exclude that other hepatic genes related to VLDL export account for the different plasma TG concentrations.

### Effect of AEA on energy balance, hepatic and mammary gene expressions in week 4 and 5 postpartum

Unexpectedly, AEA treated cows stopped increasing feed intake in week 4 p.p, while feed intake, as expected, further increased in the CON group. The reduced feed intake in the AEA group was also reflected by the lower milk protein percentage as previous studies showed a decrease in milk protein content during restricted feeding^52^. Moreover, the insufficient feed intake of the AEA group was also accompanied by a higher proportion of the lipid droplet area of the liver, indicating a higher lipid accumulation in the AEA group.

In rodents and cows, AEA administrations either increased or did not affect short-term feed intake^14,16,31,53^, but there are no previous studies reporting a decrease in short-term feed intake in mammals. Here we report that long-term administration of AEA decreases feed intake of cows after 4 weeks of treatment. One possible explanation for the reduction in feed intake could be that the long treatment duration induced a contra-regulatory mechanism in which plasma leptin concentrations increased in AEA cows in week 4 p.p. In rodents, humans and ruminants, leptin decreases feed intake and controls energy balance^11–13,54–56^. Because plasma leptin concentrations are usually determined by the amount of stored triglycerides^57^, a negative energy balance and fat mobilisation results in a decrease in plasma leptin concentration^58^. However, in our study we found that the AEA group had a similar BCS throughout the experimental period and a similar amount of adipose tissue as the CON group, except for minor differences in omental adipose tissue in week 2 and 3 p.p. These facts cannot explain the abrupt increase in leptin concentration in week 4 p.p., and indicates that plasma leptin concentrations do not correspond to adipose tissue mobilisation.

There is evidence that leptin can modulate the AEA level in non-ruminants^10,59^. Hence, it is conceivable that the increase in plasma leptin concentration in week 4 p.p. could be a counter-regulatory response to the chronic AEA administrations. However, no study has proved this hypothesis and thus further studies are needed to elucidate the interaction between the AEA tone and leptin release.

In the liver, the *FAAH* mRNA abundance was higher in the AEA group than in the control group on d 30 p.p. *FAAH* encodes the enzyme responsible for the degradation of AEA^60^ and thus, upregulation of *FAHH* implies increased degradation of AEA. Unfortunately, we were not able to measure the AEA concentration on d 30 p.p. to support this assumption. Previous studies reported increased *FAAH* mRNA abundance after AEA administration^61^. However, another reason for the change in *FAAH* mRNA abundance could be the higher leptin concentration in the AEA group. In rodents, it has been shown that i.p. administration of leptin increased *FAAH* activity and thus AEA hydrolysis in the hypothalamus, however, *FAAH* gene expression was unchanged and the authors proposed a post-translational mechanism increasing FAAH activity^59^. From these findings, we conclude that upregulation of the *FAAH* mRNA abundance was either triggered directly by repeated administration of AEA or indirectly via leptin.

The analysis of mRNA of genes related to lipid metabolism revealed a tendency to a lower *MTTP* mRNA abundance in the AEA group on d 30 p.p. The MTTP is involved in apolipoprotein assembly and the export of TG from the liver^62^. Bremmer et al. reported a trend for a negative correlation between *MTTP* mRNA expression and liver TG concentration on d 35 p.p.^64^. Similarly, cows with higher liver fat concentrations had a reduced *MTTP* mRNA expression level compared to controls^65^. In the present study, *MTTP* downregulation was also accompanied by higher lipid accumulation, as reflected by the trend to greater lipid droplet area in the liver of AEA cows; but whether AEA or the liver fat content regulates *MTTP* expression is not known. Furthermore, the biological significance of an altered *MTTP* expression remains elusive, because a change in *MTTP* mRNA abundance is not necessarily accompanied by a change in MTTP activity^63^. The higher lipid accumulation on day 30 was probably also the result of the lower feed intake and the numerically higher plasma NEFA concentration of the AEA group. Another possible explanation could be that AEA administration directly promoted hepatic fat accumulation, as CB1 activation stimulated the expression of lipogenic genes in the liver of mice^20^. However, in our study the expression of genes involved in lipid metabolism was not altered on day 30 p.p., except for *MTTP.*

As mentioned above, activation of the ECS regulates lipid metabolism in the liver and adipose tissue of rodents and ruminants, and involves among others upregulation of *ACC1* in lipogenic tissues^20^. However, little is known about the influence of endocannabinoids on the metabolism of the mammary gland. In the present study, AEA cows have a lower mammary gland *ACC1* mRNA abundance, which is involved in de novo milk fatty acid synthesis^66^. Surprisingly, downregulation of *ACC1* was not accompanied by a reduction in milk fat content. Perhaps the de novo milk fat synthesis from acetate and butyrate was inhibited and milk fat concentration was maintained due to increased uptake of long-chain fatty acids from the circulation. However, the effect of AEA on milk fatty acid composition needs to be evaluated in future studies. Downregulation of *ACC1* could also be due to higher leptin concentrations in AEA cows on d 28 p.p. Leptin inhibits ACC1 by activating AMP-activated protein kinase^67,68^, however, whether this pathway is also activated in the mammary gland of AEA cows requires further investigations. Furthermore, the decreased PLTP activity may be a result of greater leptin concentrations in AEA cows on d 28 p.p. The PLTP mediates the transfer of phospholipids to high-density lipoprotein cholesterol ^69^, however, high-density lipoprotein cholesterol plasma concentration was unchanged in our study. Nonetheless, our result corresponds to the finding in heterozygous PLTP^+/–^ mice, which had reduced PLTP activity abut no change in high-density lipoprotein cholesterol level compared to the wildtype^70^.

### Effect of AEA on whole-body energy metabolisms in week 4 postpartum

Relative to the CON group, AEA cows showed a higher postprandial FOX and lower postprandial COX. In addition, AEA treated cows tended to or had higher cumulative feed intake beginning 3 h after feeding. Because feed intake is negatively and FOX positively correlated with COX^71^, the observed differences in whole-body energy metabolism could be related to the different cumulative feed intake between groups. Our results are in contrast to a previous study in late-lactating cows, in which AEA administration increased short-term feed intake, metabolic heat production, and COX, while decreasing FOX^15^. These contrasting findings suggests that the effect of AEA administration clearly depends on the energy balance differing between stages of lactation. The AEA treatment in late-lactating cows supports anabolism^15^, whereas, as shown herein, it induces whole-body catabolism and increases lipolysis in early-lactating cows. However, the observed differences in cumulative feed intake and metabolism between AEA and CON early-lactating cows may not be attributed to the AEA administration itself, but be overridden by the higher plasma leptin concentrations. In contrast to AEA, leptin exerts catabolic effects, reduces feed intake^11^, and increases fat oxidation^68^.

### Comparative analysis of the AEA effect in different stages of lactation

Myers et al. proposed that the sensitivity to endocannabinoids varies in the adipose tissue due to the physiological status^27^. However, a lactation stage-dependent sensitivity to ECS activation could also be present in other tissues. In mid- and late-lactating cows, AEA administration increased short-term feed intake (1–10 h) but had no effect on total daily feed intake^15,28^, probably due to the short half-life of AEA. If the short AEA half-life affected the results of the present study remains questionable.

In early-lactation, AEA administration also showed no positive effect on total daily feed intake, but, this may be due to the short half-life of AEA or the dominating role of leptin. Nevertheless, endocannabinoid concentrations were found to directly correlate with an increase^28^ or decrease^72^ in feed intake during early-lactation, suggesting their involvement in the regulation of feed intake when not disturbed by leptin. When cows in late lactation are treated with AEA, they respond with a reduction in plasma NEFA concentration and thus lower lipomobilisation^15^. In contrast, AEA administration did not reduce lipomobilisation in early-lactation. Consistent with in vitro studies, CNR1 activation did not alter lipolysis rate in adipose tissue explanted from periparturient cows, whereas the lipolysis rate was reduced in adipose tissue collected from non-lactating and non-gestating cows^27^.

In the liver, we observed downregulation of the mRNA of genes related to TG synthesis, which may lead to less lipid accumulation in this organ. In contrast, in late-lactating dairy cows, AEA administration did not affect the mRNA abundance of genes involved in fat metabolism^15^. These results suggest a tissue-specific sensitivity to ECS activation depending on the physiological status of the cows, as proposed by Myers et al.^27^. However, further research is needed to elucidate the underlying mechanisms.

In conclusion, the present study shows that repeated AEA administration in the first three weeks p.p. did not affect feed intake, energy balance, milk yield or milk composition. Furthermore, repeated AEA administration did not alter lipomobilisation. However, a three-week AEA treatment affected TG synthesis in the liver, underscoring a tissue-specific AEA insensitivity in early-lactation. Chronic elevation of the AEA level after 4 weeks of administration may resulted in a counter regulatory leptin increase, which coincided with a reduction in feed intake and consequently a higher hepatic lipid accumulation, increased whole-body fat oxidation and lower whole-body carbohydrate oxidation. Further investigations are needed to understand the interaction between leptin and AEA in early-lactation.

## Material and methods

### Animals and housing

The experimental protocol was approved by the Federal Office of Agriculture, Food Security and Fishery Mecklenburg-Western Pomerania, Rostock, Germany (LALLF, permission no. 7221.3-1-015/19) and conducted in accordance with the ARRIVE guidelines (https://arriveguidelines.org/), the European Directive 2010/63/EU, the German Animal Welfare Act and the German Regulation on the Protection of Animals in Connection with Slaughter or Killing and on the Implementation of Council Regulation (EC) No 1099/2009. All persons involved in this study were blinded, except the persons who did the i.p. injections, the ultrasound measurements, the biopsies and the blood sampling.

For this study, 20 German Holstein cows at the end of their 1st (n = 12) or 2nd (n = 8) lactation were purchased from a local farm (Agrarprodukte Dedelow GmbH, Dedelow, Germany; the farm has consented to the use as experimental animals) in 10 blocks of 2 cows. Cows of each pair were half sibs, were in the same lactation number, but differed in age (± 5.5 months) and estimated calving day (± 6 d). Half sib pairs were chosen to reduce the genetic variance known to influence fat metabolism, milk yield and feed intake^73–76^. Pairs were transferred on d 56 (± 18 d) before expected calving to the free stall barn of the Experimental Facility for Cattle (Research Institute for Farm Animal Biology, Dummerstorf, Germany). Animals were habituated the respirations chambers on 3 different days between day -53 and day -23 before expected calving date. The duration of stay in the chambers was successively increased from initially 2 h to 8 h per day. Animals were considered habituated to the respirations chambers when they consumed feed and water, laid down, and ruminated.

### Feeding and milking

Cows were dried-off on d 51 (± 24 d) before the estimated calving date (except one cow on d 137 before expected calving). During the dry period, cows received a far-off diet until d 25 (± 8 d) before expected calving, following a close-up diet from d 24 (± 8 d) until parturition. After parturition, cows received a lactation diet (Table 3). All diets were offered as total mixed ration. Cows had ad libitum access to water and feed, except between 0500 and 0745 h for reasons of maintenance and to synchronize the start of feed intake after the morning feeding. Feed samples were taken weekly and the dry matter content was determined by drying samples for 24 h at 60 °C and subsequently for 4 h at 103 °C. Nutrient composition was analysed by the Landwirtschaftliche Untersuchungs- und Forschungsanstalt (LUFA GmbH, Rostock, Germany) using near infrared spectroscopy according to VDLUFA (2004) (Table 3). The individual daily feed intake as measured by the Roughage Intake Control system (RIC, Insentec B. V., Marknesse, The Netherlands) was used to calculate a weekly mean.

**Table 3:** Feed constituents, nutrient composition and energy content of far-of, close-up and lactation diet (mean and SD).

	Far-off		Close-up		Lactation	
	mean	SD	mean	SD	mean	SD
Component, g/kg of DM						
Grass/alfalfa-silage	514.9	64.5	246.4	26.4	214.1	19.9
Corn silage	104.5	24.1	352.4	12.4	376.2	10.3
Rye/triticale silage	125.3	55.9	28.7	13.6	13.2	5.8
Hay	78.0	10.2	66.9	6.7		
Straw	169.6	22.9	61.8	6.2	31.9	3.1
Rapeseed extraction meal			37.4	5.6	45.7	1.7
Soybean extraction meal			32.3	10.0	21.8	3.9
Wheat meal			48.8	5.4	16.7	0.5
Corn meal					52.6	4.9
Milk performance feed^a^			111.6	10.3	217.1	4.5
Mineral feed^b^	7.7	0.2			6.4	0.2
Mineral feed^c^			13.7	0.5		
Limestone					3.4	0.3
Soybean oil					0.9	0.1
Nutrients, g/kg of DM						
Crude ash	78.2	6.1	62.0	2.2	59.8	1.6
Crude protein	133.2	2.0	148.4	1.6	165.0	4.0
Crude fat	20.4	1.3	25.8	0.3	30.7	0.4
Crude fiber	260.9	6.3	192.8	3.3	155.0	2.7
Starch	90.5	6.0	217.4	6.0	273.2	8.3
Sugar	7.4	4.0	17.0	3.2	22.8	2.6
DM, %	33.6	0.6	38.7	0.6	40.8	0.4
ME, MJ/kg DM	8.5	0.2	10.5	0.1	11.4	0.1
NE_L_, MJ/kg DM	4.9	0.1	6.3	0.1	7.0	0.0

^a^MF2000 (Ceravis AG, Rendsburg, Germany): Soybean extraction meal from hulled seed; steam-heated, wheat, corn, canola extraction meal, beet molasses pulp, malt germ, dried stillage (grain), beet molasses, sodium bicarbonate, beet vinasse, calcium carbonate, sodium chloride, calcium-sodium phosphate, 24% crude protein, 2.6% crude fat, 5.1% crude fiber, 8% crude ash, 0.73% calcium, 0.5% phosphorous, 0.65% sodium, 7.1 MJ NEL/kg; Additives per kg organic matter: 10,000 I.U vitamin A, 1125 IU vitamin D, 40 mg vitamin E, 0.6 mg I, 0.4 mg Co, 50 mg Mn, 75 mg Zn, 0.4 mg Se.

^b^Panto Mineral R 8609 (HL Hamburger Leistungsfutter GmbH, Hamburg, Germany): 20% calcium, 6% phosphorous, 8% sodium, 6% magnesium, 0.03% inorganic nitrogen, 13.7% phosphorous pentoxide; Additives per kg original substance: 900,000 IU vitamin A, 200,000 IU vitamin D3, 4500 mg vitamin E, 1.5 g Cu, 8 g Zn, 5 g Mn, 60 mg I, 21 mg Co, 50 mg Se.

^c^KULMIN MFV Plus (Bergophor Futtermittelfabrik Dr. Berger GmbH & Co. KG, Kulmbach, Germany): 0.7% calcium, 5.5% phosphorous, 10% magnesium, 5% sodium, 3.5% HCl-insoluble ash; additives per kg organic matter: 850,000 IU vitamin A, 200,000 IU vitamin D3, 8000 mg vitamin E, 200 mg vitamin B1, 80 mg vitamin B2, 100 mg vitamin B6, 25.000 mg vitamin B12, 200 mg vitamin B5, 1000 mg niacin amide. 100,000 mg biotin, 10,000 mg choline chloride, 1000 mg Cu, 5000 mg Zn, 3000 mg Mn. 20 mg Co, 75 mg I, 45 mg Se. 14.0 *10^19 CFU Saccharomyces cerevisiae, 75 mg propyl gallate, tocopherol excract of plant oils, citric acid, 40,000 mg flavoring blend with 8.5% polyphenol content.

After calving, cows were milked twice daily at 0500 h and 1630 h and the milk yield was recorded. Milk composition was analysed by infrared spectroscopy (MilkoScan; Foss GmbH, Hillerød, Denmark) at the State Inspection Association for Performance and Quality Testing Mecklenburg-Western Pomerania e.V. (LKV Güstrow, Germany). For this purpose, milk samples from the evening and morning milking were pooled once a week. The ECM yield was calculated according to the GfE (2001)^77^:

$$ {\text{ECM }}\left( {{\text{kg}}/{\text{d}}} \right) = {\text{milk}}\;{\text{yield}}\;\left( {{\text{kg}}/{\text{d}}} \right) \times \left( {\left( {{1}.0{5} + 0.{38} \times {\text{milk}}\;{\text{fat}}\,\% + 0.{21} \times {\text{milk}}\;{\text{protein}}\,\% } \right)/{3}.{28}} \right). $$

The energy balance (EB) was calculated according to GfE (2001)^77^ as follows:

For antepartum period:

$$ {\text{EB}}_{{{\text{antepartum}}}} \left( {{\text{MJ}}\;{\text{of}}\;{\text{NE}}_{{\text{L}}} {\text{/d}}} \right) = {\text{Net}}\;{\text{energy}}\;{\text{of}}\;{\text{lactation}}\;\left( {{\text{NE}}_{{\text{L}}} } \right)\;{\text{intake}}{-}\left( {{\text{NE}}_{{\text{L}}} \;{\text{pregnancy}} + {\text{NE}}_{{\text{L}}} \;{\text{maintenance}}} \right) $$

For postpartum period:

$$ {\text{EB}}_{{{\text{postpartum}}}} = {\text{NE}}_{{\text{L}}} {\text{intake}}{-}\left( {{\text{NE}}_{{\text{L}}} {\text{maintenance}} + {\text{NE}}_{{\text{L}}} {\text{milk}}} \right). $$

NE_L_ intake, NE_L_ maintenance, NE_L_ pregnancy, and NE_L_ milk were calculated as follows:

$$ \begin{aligned} & {\text{NE}}_{{\text{L}}} \;{\text{intake}} = {\text{DMI}} \times {\text{NE}}_{{\text{L}}} \\ & {\text{NE}}_{{\text{L}}} \;{\text{maintenance}} = 0.{293} \times {\text{mBW}} \\ & {\text{NE}}_{{\text{L}}} \;{\text{pregnancy}} = 0.0{44} \times {\text{e}}^{{0.0{165}\; \times \;{\text{day}}\;{\text{of}}\;{\text{conception}}}} \\ & {\text{NE}}_{{\text{L}}} \;{\text{milk}} = {\text{ECM}} \times {3}.{28} \\ \end{aligned} $$

### Body weight, BCS and ultrasound measurements

Body weight (BW) was recorded once a week in the a.p. period and twice daily in the p.p. period after milking using a walk-through scale. From the weekly mean, the mBW was calculated. Once a week, the BCS was determined according to Edmonson et al.^78^ and the heart girth was measured according to Branton and Salisbury^79^. The back fat thickness was measured according to Staufenbiel^80^ and the subcutaneous fat layer over the 12th rib according to Raschka et al.^81^, each once a week via ultrasound. Additional ultrasound measurements were taken to calculate the amount of subcutaneous adipose tissue, retroperitoneal adipose tissue , omental adipose tissue and mesenteric adipose tissue according to Raschka et al.^81^. The total abdominal adipose tissue was calculated as the sum of retroperitoneal, omental and mesenteric adipose tissue. The devices and ultrasound probes used for the respective ultrasound measurements are shown in Supplementary Table 1.

### Treatments

Each cow of the half-sib pair was randomly assigned to either a treatment group (AEA, n = 10) or control group (CON, n = 10). Treatment started on d 1 after parturition if the cow calved before 1400 h or on d 2 if the cow calved after 1400 h. The AEA group received 3 µg/kg BW/d of *N*-arachidonoylethanolamine (AEA; Tocris, Bristol, UK) diluted in 50 mL 0.9% NaCl via i.p. injections. The i.p. injections were administered at the right paralumbar fossa as described previously by van Ackern et al.^14^. We chose i.p. injection because it allows rapid AEA absorption into the systemic circulation and the activation of the splanchnic ECS and vagal afferents of the gut-brain-axis^15^.

The administered AEA dose remained constant during the treatment period and was calculated based on the BW determined at the d of calving. The CON group received 50 mL of 0.9% NaCl i.p. Injections were administrated as daily bolus from Mondays to Fridays at 0700 h (± 34 min) until d 30 (± 1 d) p.p. Due to logistic reasons, administrations interrupted on Saturdays and Sundays.

### Blood sampling and analyses

Blood samples were collected from the jugular vein on d -10 (± 2 d), + 1 (± 1 d), + 3 (± 1 d), + 8 (± 1 d), + 14 (± 2 d), + 21 (± 1 d) and + 28 (± 4 d) relative to parturition at 0745 h in EDTA-containing tubes (Sarstedt AG & Co. KG, Nümbrecht, Germany) and were immediately placed on ice. Subsequently, blood samples were centrifuged at 1570 × *g* for 20 min at 4 °C. The obtained plasma was stored at − 80 °C for further analysis. All analyses were performed in single measurements, except for leptin which was analysed in duplicates.

Plasma concentrations of NEFA (Kit: NEFA-HR (2), FUJIFILM Wako Chemicals Europe GmbH, Neuss, Germany), 3-hydroxybutyric acid (D-3-Hydroxybutyrate Ranbut assay, RANDOX, Crumlin, United Kingdom), glucose (ABX Pentra Glucose HK CP, HORIBA ABX, Montpellier, France), urea (ABX Pentra Urea CP, HORIBA ABX), triglycerides (Kit: ABX Pentra Triglycerides CP, HORIBA ABX,) and high density lipoprotein cholesterol (Kit: ABX Pentra HDL Direct CP, HORIBA ABX,) were analysed using kits at a semi-automatic spectrophotometer (ABX Pentra 400, HORIBA Medical, Kyoto, Japan). The variation coefficients for these kits were 1.50%, 1.17%, 0.70%, 2.27%, 2.52%, and 2.51%, respectively.

The plasma concentration of the endocannabinoids AEA, 2-arachidonoylglycerol (2-AG), eicosapentaenoyl ethanolamide (EPEA), docosahexaenoyl ethanolamide (DHEA) as well as the non-endocannabinoids *N*-acylethanolamines palmitoylethanolamide (PEA), oleoylethanolamide (OEA), and linoleoyl ethanolamide (LEA) was measured in plasma samples collected on d − 10 (± 2 d), + 8 (± 1 d), + 14 (± 2 d) and + 21 (± 1 d) relative to parturition by LIPIDOMIX GmbH, Berlin, Germany using a triplequad mass spectrometer coupled HPLC. The quantitation limit was 0.01 ng/mL and the detection limit 0.003 ng/mL. The recovery rate ranged between 80 and 110%. Results were corrected for recovery.

Plasma samples collected on d -10 (± 2 d), + 3 (± 1 d), + 8 (± 1 d), + 14 (± 2 d), + 21 (± 1 d) and + 28 (± 4) relative to parturition were used to determine phospholipid transfer protein (PLTP) activity using a commercial fluorescence activity assay (MAK108 Kit, Roar Biomedical Inc., New York, NY, USA).

Plasma leptin concentrations were measured in duplicates in samples collected on d − 10 (± 2 d), + 8 (± 1 d), + 14 (± 2 d), + 21(± 1 d) and + 28 (± 4 d) with an enzyme immunoassay by the Institute of Animal Sciences, Physiology and Hygiene, Bonn University, Bonn, Germany as described by Sauerwein et al.^82^. Intra-assay and inter-assay coefficients of variation were 8.63% and 10.22%, respectively.

### Subcutaneous fat biopsy and liver biopsy

On d 25 (± 10 d) before expected calving and on d 16 (± 2 d) p.p. a liver biopsy and a subcutaneous adipose tissue biopsy were taken. The liver was scanned using ultrasound [L52x Rectal Transducer (10-5 MHz), Fujifilm SonoSite Inc., Bothell, WA; SonoSite MicroMaxx; Fujifilm SonoSite Inc., Bothell, WA; USA] to locate the insertion site and avoid injuries to large hepatic blood vessels or the intestine. A 15 × 15 cm area located approximately 10 cm below the *processus transversi* was shaved, washed, disinfected, and 10 mL of 2% procainhydrochlorid (Procamidor, WDT, Garbsen, Germany) were injected subcutaneously and into the muscles of the 12th, 11th, or 10th intercostal space. After stab incision in the respective intercostal space with a scalpel, liver tissue was obtained using the Pro-Mag Ultra 2.2 Liver device (Plano, USA) with a 13 gauge needle (1st and 2nd block) or a tailor-made two-part trocar needle with an outer diameter of 6 mm (3rd to 10th block). Liver tissue samples were immediately snap frozen in liquid nitrogen and stored at − 80 °C until analysis.

For the adipose tissue biopsy, cows received epidural anaesthesia with 5 mL of 2% procainhydrochlorid (Procamidor, WDT, Garbsen, Germany). Furthermore, 10 mL procainhydrochlorid were subcutaneously injected above the sacrotuberal ligament. After shaving, cleaning and disinfection, the skin of the ischiorectal fossa was cut and adipose tissue was taken through a 2.5 cm long incision. It was not possible to obtain subcutaneous adipose tissue from one CON cow p.p.

The incisions were sutured with non-absorbable surgical suture (DERMAFIL GREEN, SMI AG, St.Vith, Belgium) and covered with aluminium spray (Aluminium Spray, Pharmamedico GmbH, Twistringen, Germany). Stitches were removed after 10 d.

Immediately after collection, half of the harvested adipose tissue was frozen in liquid nitrogen and stored at − 80 °C for further analysis. The other half of the obtained adipose tissue biopsy was used to determine in vitro lipolysis according to Kokkonen et al.^83^. Briefly, biopsy tissue was immersed in 37 °C Krebs–Ringer solution (Thermo Fisher Scientific Inc., Waltham, USA) supplemented with 15 mmol/L NaHCO_3_ and 2.5 mmol/L CaCl_2_, and transported within 10 min to the laboratory. The tissue was cut into 5 mm pieces. Approximately 250 mg tissue were incubated in 3 mL of the medium mentioned above but saturated with O_2_ at 37 °C. After 15 min of incubation, 1 mL of the incubation medium was removed and frozen at − 20 °C. Ten µl of a solution containing 10 mM noradrenaline-hydrochloride and 2 M glucose were added to the remaining medium before the medium was gassed with O_2_ and incubated for 120 min at 37 °C. Thereafter, 1 mL medium was taken and frozen at − 20 °C and the fat tissue subjected to freeze-drying to determined tissue dry matter. The 1-mL media samples were thawed and the dissolved protein precipitated by adding 0.9 mL acetonitrile to 0.3 mL medium. After mixing and centrifugation at 13,000 g and 4 °C for 20 min, the supernatant was evaporated to dryness and re-dissolved in ultra-pure water by sonication with a concentration factor of 2 and centrifuged again for 10 min. The glycerol concentrations were measured using HPLC with a refractive index detector (1200/1260 infinity Series, Agilent Technologies). Chromatographic separation of 50 µL solution was carried out on a 300 × 7.8 mm Rezex ROA-Organic Acid H + (8%) column (Phenomenex, Aschaffenburg, Germany) protected with a 4 × 3 mm Carbo-H + guard cartridge (Phenomenex, Aschaffenburg, Germany) at 75 °C using sulphuric acid (5 mM) as eluent with a flow rate of 0.4 mL/min. Calculations were done by the use of external standards from 0.05 to 0.5 mmol/L and results were corrected for recovery. The lipolysis rate was calculated by subtracting the glycerol concentration at the time t = 15 min from the concentration at t = 120 min.

### Indirect calorimetry

On d 27 (± 2 d) p.p., each cow was transferred into one of four open-circuit respiration chambers and individually kept in tie-stalls at 15 °C until d 29 (± 2 d). The BW was recorded before and after entering the respiration chamber. Milking was performed at 0630 h and 1700 h, and the milk yield was recorded. Cows received the i.p. administrations as described above and were fed 45 min post administration.

The 24-h gas concentration measurement started after over-night gas equilibration on d 28 (± 2 d) at 0700 h. CO_2_, O_2_, CH_4_ concentrations and feed intake were measured in 6-min intervals as described by Derno et al.^84^. The airflow through the chamber was set to approximately 30 m^3^/h and was measured by a differential pressure type V cone flow meter (McCrometer, Hemet, CA). The mean CO_2_ recovery rate of each chamber was 99.9%.

Total CO_2_ production is composed of CO_2_ from fermentative (CO_2_ ferm) and metabolic (CO_2_ metab) processes. CO_2_ ferm was estimated according to Chwalibog et al. as CO_2_ ferm (L) = 1.7 × CH_4_ (L)^85^. Metabolic CO_2_ was calculated by subtracting CO_2_ ferm from total CO_2_.

Due to technical issues, only the data obtained before feeding and up to 7 h after feeding were considered in the following calculations:

The heat production (HP) was calculated according to Brouwer^86^:

$$ {\text{HP}}\;\left( {{\text{kJ}}} \right) = {16}.{18}*{\text{O}}_{{2}} \left( {\text{L}} \right) + {5}.0{2}*{\text{CO}}_{{2}} {\text{total}}\;\left( {\text{L}} \right) - {2}.{17}\;{\text{CH}}_{{4}} \left( {\text{L}} \right) - {5}.{99}\;{\text{N}}_{{\text{u}}} \left( {\text{g}} \right). $$

Net carbohydrate oxidation (COX) and net fat oxidation (FOX) were calculated according to Frayn^87^:

$$ {\text{COX}}\;\left( {\text{g}} \right) = {4}.{55}*{\text{mCO}}_{{2}} \left( {\text{L}} \right) - {3}.{21}*{\text{O}}_{{2}} \left( {\text{L}} \right) - {2}.{87}*{\text{N}}_{{\text{u}}} ({\text{g}}). $$

$$ {\text{FOX}}\;\left( {\text{g}} \right) = {1}.{67}*{\text{O}}_{{2}} \left( {\text{L}} \right) - {1}.{67}*{\text{mCO}}_{{2}} \left( {\text{L}} \right) - {1}.{92}*{\text{N}}_{{\text{u}}} \left( {\text{g}} \right). $$

Urinary N excretion (N_u_) was estimated to 100 g/d, considering real N_u_ values ranging from 75 to 150 g/d^88^. Thereby, an error of less than 5% of HP, COX and FOX was accepted.

Data for HP, FOX and COX were normalized to mBW. Changes in FOX, COX and HP, as well as cumulative DMI were calculated in relation to the start of feeding (0745 h) and evaluated in hourly intervals.

### Slaughter and quantitative real-time-PCR (RT-qPCR)

On d 30 (± 1 d) p.p., cows were sacrificed 1–1.5 h after AEA or CON administration by captive bolt stunning and subsequent exsanguination at the institutional abattoir. Samples were taken from the lobus quadratus of the liver and from the right rear quarter of the mammary gland, snap frozen in liquid nitrogen, and stored at − 80 °C for further analysis. These tissue samples as well as the liver samples obtained from the biopsies were ground in a mortar under liquid nitrogen. Approximately 18 mg powdered tissue were extracted, respectively, for total RNA using the innuPREP RNA Mini Kit 2.0 (AJ Innuscreen GmbH, Berlin, Germany). The RNA samples were DNA digested (innuPREP DNase I Digest Kit, AJ Innuscreen GmbH) and the RNA quality assessed with an Agilent 2100 Bioanalyzer (Agilent Technologies Inc., Santa Clara, CA, USA) yielding RNA integrity numbers (RIN) between 6.4 and 9.1 (mean: 8.3, SD: 0.6). For first-strand cDNA synthesis, 1 µg RNA was reverse transcribed (SensiFast™ cDNA Synthesis Kit; Bioline, London, UK) using a Thermocycler (peqstar 96 × HPL, VWR International, Pennsylvania, USA).

Used primers are shown in Supplementary Table 2 and 3. The qPCR was performed in duplicates on a Light Cycler^®^ 96 (Roche, Basel, Switzerland). One PCR reaction contained 2 µL cDNA (10 ng/µL), 1 µL H_2_O, 0.5 µL of each primer (4 µM), and 6 µL 2 × Puffer SensiFAST SYBR No-ROX mix (Bioline, London, UK). The amplification efficiency was calculated with LinRegPCR software (Version 2014.4; Academic Medical Centre, Amsterdam, The Netherlands) Data was quantified by qbasePlus software (Biogazelle, Gent, Belgium). Eukaryotic translation initiation factor 3 subunit k (EIF3K) and peptidylprolyl isomerase A (PPIA) were used as reference genes for the analysis of the mammary gland (M-value: 0.257 CV-value: 0.089). Emerin (EMD) and EIF3K was used as reference genes for the liver tissue (M-value: 0.255 CV-value: 0.089).

### Liver fat

Frozen liver tissue samples taken after slaughter were cut into 6-μm sections with a cryostat microtome (CM3050 S, Leica, Bensheim, Germany) and stained with Oil Red O (Chroma Gesellschaft, Münster, Germany). Nine randomly selected images (total area 3.3 mm^2^) per cow were taken, except for one animal for which 8 images were taken (total area 2.9 mm^2^), using an Olympus BX43 microscope (Olympus, Hamburg, Germany) equipped with a UC30 colour camera (OSIS, Münster, Germany) and cellSens image analysis software (Evident, Hamburg, Germany). The lipid area was identified using a colour threshold operation and the number of lipid droplets and the lipid droplet area were determined using the “Count and Measure” function of the cellSens software. In addition, the number of lipid droplets/mm^2^ and the percentage of lipid droplet area of the total measured area were calculated.

### Statistical analyses

The sample size required was calculated with CADEMO for Windows (ANOV version 4.03, BioMath GmbH, Rostock) using ANOVA-F-Tests, assuming a biological relevance of at least 1.4 residual standard deviations, and a probability for the type I error of α = 0.5 and type II error of β = 0.2.

All statistical evaluations were performed using R Statistical Software (v4.2.0; R Core Team 2021, R Foundation for Statistical Computing, Vienna, Austria). Outliers were detected by using cooks distance (olsrr package, v0.5.3; Hebbali 2020) and visual inspection of boxplots. Two outliers in the dataset of AEA plasma concentration on d + 14 were detected and excluded from the subsequent statistical analysis. Furthermore, one outlier was detected and excluded in the dataset of mRNA analysis of hepatic genes (*CNR1, GPR55, DGAT1/2* in liver tissue obtained on d -25 and on d 30). Due to technical problems, two animals were excluded from the indirect calorimetry analysis. In addition, one animal was excluded from the fat depot analyses, because it had haematomas at the measurement sites. Another animal was excluded from all analyses in week 4 and 5 p.p. due to intestinal obstruction.

Data were analysed with a linear mixed model (LMM, lmer function, lme4 package, v1.1–29; Bates, Maechler, Walker 2015^89^). For the analysis of gene expressions, the plasma concentration of endocannabinoids, *N*-acylethanolamines, plasma metabolites, leptin., PLTP activity a.p. and the analysis of the number of lipid droplets and lipid droplet area in liver tissue the model contained “group” (level: AEA and CON) as fixed effect and “sire” as random effect. The same model was used for dataset of lipolysis rate a.p. and p.p., respectively. To evaluate the plasma concentration of endocannabinoids, N-acylethanolamines, plasma metabolites, leptin, PLTP activity p.p. and data obtained from indirect calorimetry, the model contained the fixed effects “group” (level: AEA and CON), “time” and the interaction (group x time), with “time” as repeated variable. Additionally, the model contained “sire” and “cow ID” as random effects. The equal model was used to evaluate the data of the BW, DMI/mBW, energy balance, milk yield, milk constituents and data obtained from ultrasound measurements, whereby data were considered separately for a.p. and p.p. period. The fixed effect "lactation number" was tested but did not improve the models. All models were tested for homoscedasticity and normal distribution of the residuals (check_normality and check_heteroscedasticity function, performance package, v0.9.0; Lüdecke et al. 2021^90^). Heteroscedasticity has only a marginal impact on model estimates^91^, allowing the models to be used despite the violation of the constant error variance. If the assumption of normality was violated, data were transformed with the Johnson transformation. In case the transformation was not successful, a generalized linear mixed model with a gamma distribution was used. The Wilcoxon signed rank test was used to analyse EPEA during a.p. period, because no normal distribution of the residuals could be achieved after transformation and gamma distribution did not fit.

Pairwise differences between levels of fixed effects were tested by using the Tukey Kramer test. For the fixed effect of interest, estimated marginal means and their standard errors (SEs) were estimated. Effects and differences were considered significant at *P* < 0.05 and as a trend at *P* < 0.1. For interpretation purposes, the means of the observed data and their standard deviations (SDs) are presented in the figures and tables.

### Supplementary Information

Supplementary Information.

## Data Availability

All data generated and analysed are available on request from the corresponding author.
