# Supplementary material for: The effect of N-arachidonoylethanolamide administration on energy and fat metabolism of early lactating dairy cows
Source: Sci Rep. 2023 Sep 6;13:14665. doi: 10.1038/s41598-023-41938-0 (PMC10482912; doi:10.1038/s41598-023-41938-0)
Supplement: Supplementary file 1 — Supplementary Information. [file 41598_2023_41938_MOESM1_ESM.docx]

**Supplemental material**

Schwerdtfeger et al. The effect of N-arachidonoylethanolamide administration on energy and fat metabolism of early lactating dairy cows


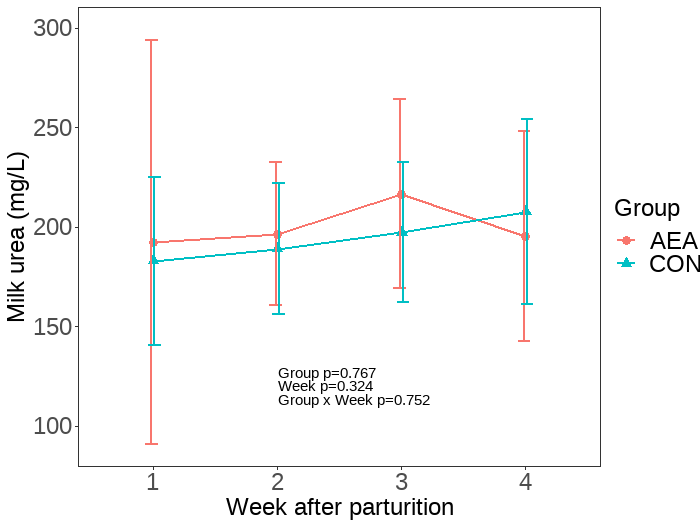


**Supplementary Figure 1.** Milk urea concentration of cows treated intraperitoneally with *N*-arachidonoylethanolamide (AEA, n = 10) or NaCl (CON, n = 10) postpartum. Data are presented as means ± SD.


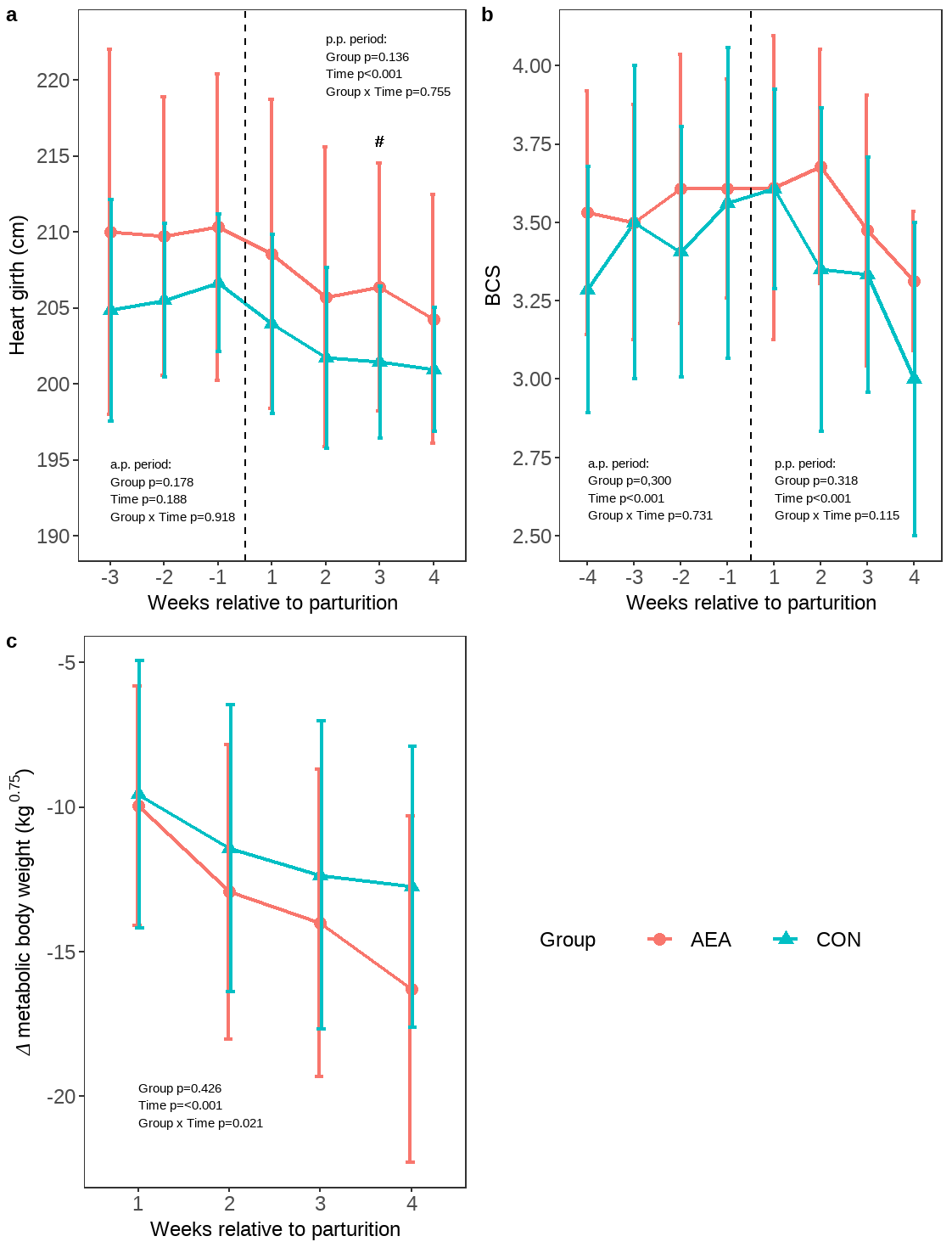


**Supplementary Figure 2.** Heart girth (**a**), body condition score (BCS; **b**), and change in metabolic body weight (**c**) of cows treated intraperitoneally with NaCl (CON, n = 10) or *N*-arachidonoylethanolamide (AEA, n = 10) postpartum. Data are presented as means ± SD; # *P* < 0.1.


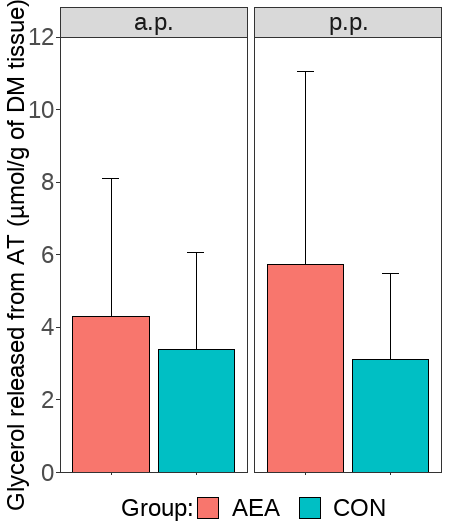


**Supplementary Figure 3.** Noradrenaline stimulated glycerol released after 120min incubation from adipose tissue (AT) of cows treated intraperitoneally with *N*-arachidonoylethanolamide (AEA, n = 8) or NaCl (CON, a.p. n = 10, p.p. n = 9 ) postpartum. Data are presented as means ± SD.

**Supplementary Table 1.**  Devices and probes used for the ultrasound measurements.

| **Ultrasound measurement** | **Calculated Fat Depot** | **Device** | **Probe** |
| --- | --- | --- | --- |
| R12 | AAT, RPAT, SCAT | SonoSite MicroMaxx; Fujifilm SonoSite Inc., Bothell, WA | L52x Rectal Transducer (10-5MHz) Fujifilm SonoSite Inc., Bothell, WA |
| BFT | OMAT, SCAT | SonoSite MicroMaxx; Fujifilm SonoSite Inc., Bothell, WA | L52x Rectal Transducer (10-5MHz) Fujifilm SonoSite Inc., Bothell, WA |
| AW1b | AAT, MAT | SonoSite MicroMaxx; Fujifilm SonoSite Inc., Bothell, WA | L52x Rectal Transducer (10-5MHz) Fujifilm SonoSite Inc., Bothell, WA |
| AW3b | OMAT, MAT | SonoSite MicroMaxx; Fujifilm SonoSite Inc., Bothell, WA | L52x Rectal Transducer (10-5MHz) Fujifilm SonoSite Inc., Bothell, WA |
| AW3c | MAT, SCAT | SonoSite MicroMaxx; Fujifilm SonoSite Inc., Bothell, WA | L52x Rectal Transducer (10-5MHz) Fujifilm SonoSite Inc., Bothell, WA |
| KD2c | AAT, MAT | SonoSite Titan; Fujifilm SonoSite Inc., Bothell, WA | C60/5-2MHz convex transducer, Fujifilm SonoSite Inc., Bothell, WA |
| KD3b | RPAT | SonoSite Titan; Fujifilm SonoSite Inc., Bothell, WA | C60/5-2MHz convex transducer, Fujifilm SonoSite Inc., Bothell, WA |

R12 = subcutaneous fat over the 12^th^ rib; BFT = back fat thickness; AW1b = the distance from the skin to the distal muscle margin above the peritoneum at the point of interception of a vertical line trough the last lumbar vertebra and a horizontal line trough the patella; AW3b = the distance from the skin to the distal muscle margin above the peritoneum at the center of the paralumbar fossa; AW3c = the thickness of the abdominal wall at the center of the paralumbar fossa; KD2c = the distance from the skin to the peritoneum in the intertransverse space directly cranial to intertransverse space where the caudal pole of the kidney is visible; KD3b = the distance from the skin to the distal kidney margin in the intertransverse space directly cranial to KD2; AAT = total abdominal adipose tissue; MAT = mesenteric adipose tissue; OMAT = omental adipose tissue; RPAT = retroperitoneal adipose tissue; SCAT = subcutaneous adipose tissue

**Supplementary Table 2.** Primer sequences, PCR product sizes and amplification efficiency for genes analysed in liver tissue.

| **Gene** | **Function** | **Primer sequence (5´to 3´)** | **Accession ID** | **Size (bp)** | **Efficiency**  **(day -25/+16/+30)** | **Reference** |
| --- | --- | --- | --- | --- | --- | --- |
| *EIF3K* | Forward | CCAGGCCCACCAAGAAGAA | NM_001034489 | 125 | 1.81/1.81/1.79 | 1 |
|  | Reverse | TTATACCTTCCAGGAGGTCCATGT |  |  |  |  |
| *EMD* | Forward | GCCCTCAGCTTCACTCTCAGA | NM_203361 | 100 | 1.88/1.87 | 1 |
|  | Reverse | GAGGCGTTCCCGATCCTT |  |  |  |  |
| *GPR55* | Forward | TGCTGCCTGGATGTTTTCTG | XM_024982054 | 207 | 1.81/1.8/1.82 | 2 |
|  | Reverse | TCAGCCCACCACATCAGG |  |  |  |  |
| *CNR1* | Forward | AAGCCCGCATGGACATTCGGCTGG | NM_001242341 | 79 | 1.9/1.9/1.88 | 2 |
|  | Reverse | AGCAGAGGGCCCCAGCAGAT |  |  |  |  |
| *DGAT1* | Forward | GACCCCTAACCTTTGACCCC | NM_174693 | 163 | 1.82/1.83/1.85 | 2 |
|  | Reverse | CCCAACCTCCCGCTAAGTTT |  |  |  |  |
| *DGAT2* | Forward | GAAAGGTAGGAGCACGGGTC | NM_205793 | 132 | 1.82/1.82/1.86 | 2 |
|  | Reverse | AGCCCAACACTATTCACGCA |  |  |  |  |

| **Gene** | **Function** | **Primer sequence (5´to 3´)** | **Accession ID** | **Size (bp)** | **Efficiency**  **(day -25/+16/+30)** | **Reference** |
| --- | --- | --- | --- | --- | --- | --- |
| *FAAH* | Forward | TTCCTGCCAAGCAACATACCT | NM_001099102 | 105 | 1.69/1.70/1.77 | 2 |
|  | Reverse | CACGAAATCACCTTTGAAGTTCTG |  |  |  |  |
| *NAPEPLD* | Forward | AGAGATCACAGCAGCGTTCCAT | NM_001015680 | 95 | 1.81/1.82/1.84 | 3 |
|  | Reverse | ACTCCAGCTTCTTCAGGGTCATC |  |  |  |  |
| *ABCA1* | Forward | TGGAAGAATGTGAAGCTCTCTG | NM_001024693 | 157 | 1.75/1.74/1.76 |  |
|  | Reverse | TGTACAGGCTTCAGGTCAGG |  |  |  |  |
| *APOB100* | Forward | GCCAGAGATGAAGTGCTGGA | XM_024999521 | 149 | 1.84/1.83/1.85 |  |
|  | Reverse | ATCTTGGTGGCGCTTCTTGA |  |  |  |  |
| *MTTP* | Forward | TCATCCAATGTGGATGTCGC | NM_001101834 | 267 | 1.71/1.71/1.79 |  |
|  | Reverse | GAGATTTTCTATGGCTGCTG |  |  |  |  |
| *PPARA* | Forward | CGGAAGTCCGCATTTTCCAC | NM_001034036 | 171 | 1.81/1.81/1.80 | 2 |
|  | Reverse | TCACAGAAGACAGCATCGCA |  |  |  |  |
| *SREBF1* | Forward | GCACCGAGGCCAAGTTGAAT | NM_001113302 | 148 | 1.87/1.86/1.87 | 2 |
|  | Reverse | CACCAGTCCTTCAGCGATT |  |  |  |  |

*PPIA*, peptidylprolyl isomerase A; *EIE3K*, eukaryotic translation initiation factor 3 subunit K; *EMD*, emerin; *GPR55*, G protein-coupled receptor 55; *CNR1*, cannabinoid receptor 1; *DGAT1*, diacylglycerol o-acyltransferase 1; *DGAT2*, diacylglycerol o-acyltransferase 2; *FAAH*, fatty acid amide hydrolase; *NAPEPLD*, N-acyl phosphatidylethanolamine phospholipase D; *ABCA1*, ATP binding cassette subfamily A member 1; *APOB100*, apolipoprotein B; *MTTP*, microsomal triglyceride transfer protein; PPARA, peroxisome proliferator activated receptor alpha; *SREBF1*, sterol regulatory element binding transcription factor 1

**Supplementary Table 3.** Primer sequences, PCR product sizes and amplification efficiency for mRNA analysed in mammary gland.

| **Gene** | **Function** | **Primer sequence (5´to 3´)** | **Accession ID** | **Size (bp)** | **Efficiency** | **Reference** |
| --- | --- | --- | --- | --- | --- | --- |
| *EIF3K* | Forward | CCAGGCCCACCAAGAAGAA | NM_001034489 | 125 | 1.81 | 1 |
|  | Reverse | TTATACCTTCCAGGAGGTCCATGT |  |  |  |  |
| *PPIA* | Forward | GGATTTATGTGCCAGGGTGGTGA | NM_178320 | 120 | 1.90 | 3 |
|  | Reverse | CAAGATGCCAGGACCTGTATG |  |  |  |  |
| *CNR1* | Forward | AAGCCCGCATGGACATTCGGCTGG | NM_001242341 | 79 | 1.87 | 2 |
|  | Reverse | AGCAGAGGGCCCCAGCAGAT |  |  |  |  |
| *ACC1* | Forward | TCCTGCTGCTATTGCTACTCCA | NM_174224 | 95 | 1.86 |  |
|  | Reverse | CAGTCCCCGCACTCACATAA |  |  |  |  |
| *DGAT1* | Forward | GACCCCTAACCTTTGACCCC | NM_174693 | 163 | 1.83 | 2 |
|  | Reverse | CCCAACCTCCCGCTAAGTTT |  |  |  |  |
| *DGAT2* | Forward | GAAAGGTAGGAGCACGGGTC | NM_205793 | 132 | 1.84 | 2 |
|  | Reverse | AGCCCAACACTATTCACGCA |  |  |  |  |
| *FASN* | Forward | ACCTCGTGAAGGCTGTGACTCA | NM_001012669 | 92 | 1.88 |  |
|  | Reverse | TGAGTCGAGGCCAAGGTCTGAA |  |  |  |  |
| *SCD* | Forward | CCCTTTCCTTGAGCTGTCTG | NM_173959 | 181 | 1.86 | 4 |
|  | Reverse | ATGCTGACTCTCTCCCCTGA |  |  |  |  |
| *SREBF1* | Forward | GCACCGAGGCCAAGTTGAAT | NM_001113302 | 148 | 1.89 | 2 |
|  | Reverse | CACCAGGTCCTTCAGCGATT |  |  |  |  |

*EIE3K*, eukaryotic translation initiation factor 3 subunit K; *PPIA*, peptidylprolyl isomerase A; *CNR1*, cannabinoid receptor 1; *ACC1*, acetyl-CoA carboxylase alpha; *DGAT1*, diacylglycerol o-acyltransferase 1; *DGAT2*, diacylglycerol o-acyltransferase 2; *FASN*, fatty acid synthase; *SCD*, stearoyl-CoA desaturase; *SREBF1*, sterol regulatory element binding transcription factor 1

1 Saremi, B., Sauerwein, H., Dänicke, S. & Mielenz, M. Technical note: Identification of reference genes for gene expression studies in different bovine tissues focusing on different fat depots. *Journal of Dairy Science* **95**, 3131-3138, doi:<https://doi.org/10.3168/jds.2011-4803> (2012).

2 van Ackern, I., Wulf, R., Dannenberger, D., Tuchscherer, A. & Kuhla, B. Effects of endocannabinoids on feed intake, stress response and whole-body energy metabolism in dairy cows. *Scientific Reports* **11**, 23657, doi:10.1038/s41598-021-02970-0 (2021).

3 van Ackern, I., Kuhla, A. & Kuhla, B. A Role for Peripheral Anandamide and 2-Arachidonoylglycerol in Short-Term Food Intake and Orexigenic Hypothalamic Responses in a Species with Continuous Nutrient Delivery. *Nutrients* **13**, doi:10.3390/nu13103587 (2021).

4 Gervais, R., McFadden, J. W., Lengi, A. J., Corl, B. A. & Chouinard, P. Y. Effects of intravenous infusion of trans-10, cis-12 18:2 on mammary lipid metabolism in lactating dairy cows. *Journal of Dairy Science* **92**, 5167-5177, doi:<https://doi.org/10.3168/jds.2009-2281> (2009).
